# Do return-to-work trajectories differ by mental disorder diagnosis? A register study among 37 523 Dutch workers

**DOI:** 10.5271/sjweh.4183

**Published:** 2024-10-01

**Authors:** Robèrt Vendelbosch, Corné Roelen, Josué Almansa, Ute Bültmann, Iris Arends

**Affiliations:** 1 Department of Health Sciences, Community & Occupational Medicine, University Medical Center Groningen, University of Groningen, Groningen, the Netherlands.; 2 Arbo Unie, Knowledge Institute for Work and Health, Utrecht, the Netherlands.

**Keywords:** absenteeism, longitudinal trajectory, sick leave, sickness absence

## Abstract

**Objectives:**

Return to work (RTW) of workers with mental disorders is often a process of gradually increasing work hours over time, resulting in a RTW trajectory. This study aimed to investigate 2-year RTW trajectories by mental disorder diagnosis, examining the distribution of age, sex and contracted work hours across the diagnosis-specific RTW trajectories.

**Methods:**

Sickness absence episodes diagnosed within the ICD-10 chapter V (mental and behavioral disorders) and ICD-10 Z73.0 (burnout) were retrieved from a Dutch occupational health service register, together with age, sex and contracted work hours. Sickness absence episodes due to adjustment disorders (N=25 075), anxiety disorders (N=1335), burnout (N=3644), mood disorders (N=5076), and post-traumatic stress disorders (N=2393) were most prevalent and included in latent class growth analysis (LCGA) to estimate 23-month RTW trajectories.

**Results:**

Four main RTW trajectories were identified for all mental disorder diagnoses: fast full RTW [range 82.4% (mood disorders) to 92.0% (adjustment disorders) of the study population], slow full RTW [3.5% (burnout) to 6.1% (mood disorders)], slow partial RTW [0.6% (adjustment disorders) to 1.6% (mood disorders)] and no RTW [2.2% (adjustment disorders) to 9.7% (mood disorders)]. Trajectories with a late onset of fast full RTW included higher percentages of women and lower percentages of full-time workers.

**Conclusions:**

RTW trajectories were similar for different mental disorder diagnoses although the distribution differed across diagnoses, with more partial and no RTW trajectories among workers with mood disorders. To better guide workers back to work, more knowledge is needed of factors associated with late, partial, or no RTW.

Mental disorders are among the top ten causes of years lived with disability (1). The consequences for people affected by mental disorders and society as a whole are considerable, as reflected in impaired functioning, high sickness absence and work disability (2). Workers sick-listed with mental disorders are absent from work for long periods of time, ranging from 109 days for adjustment disorders to 321 days for personality disorders (3). While ample research has focused on reducing long-term sickness absence (4), little is known about how workers sick-listed with mental disorders return to work. The transition from sickness absence back to work can be described as the return-to-work (RTW) process, moving from off-work to work re-entry and subsequently work maintenance (5). In several countries, including Austria, Denmark, Finland, Germany, Norway, Sweden, Switzerland, and The Netherlands, work re-entry consists of a gradual increase in work hours (6, 7). Such a gradual increase in work hours has been advocated to enable early work resumption, which is positively associated with full and sustainable RTW outcomes (8–10). Following the process of gradual RTW, each sick-listed worker develops an own individual RTW trajectory.

To date, only a few studies have investigated RTW trajectories of workers sick-listed with mental disorders. Danish and Dutch studies, although different in study population and sample size, found comparable trajectories with rapid full, slow full, and no or marginal RTW (11–13). In these studies, RTW trajectories were identified for the total population of workers with mental disorders. The RTW trajectories were not diagnosis-specific, ie, not stratified by mental disorder diagnosis potentially due to sample size limits. Both Hellström et al (11) and Spronken et al (12) investigated the distribution of different mental disorder diagnoses across RTW trajectories. Hellström et al did not find any differences in the distribution of anxiety, depression and bipolar disorders across RTW trajectories, which may be explained by the fact that these disorders frequently co-exist (N=283) (11). Spronken et al reported that workers with stress complaints and adjustment disorders were more likely to follow rapid RTW trajectories, while workers with mood disorders and burnout were more likely to follow slow RTW trajectories (13). Considering these results, RTW trajectories could be diagnosis-specific. On the one hand, trajectories found for the total group of workers with mental disorders may not (all) be identified within a specific disorder group. On the other hand, trajectories for a specific mental disorder diagnosis may not be identified in the total group of workers with mental disorders. For example, workers with adjustment disorders (generally characterized by mild symptoms) may follow RTW trajectories that always result in full RTW, while workers with mood disorders or burnout (generally characterized by more severe symptoms) may follow RTW trajectories with relapses (ie, a reduction in work hours after a first increase of work hours) and partial or no RTW. Studies investigating diagnosis-specific RTW trajectories are currently lacking. Understanding the RTW trajectories for specific mental disorder diagnoses could inform (occupational) health professionals whether workers with certain diagnoses are at increased risk of unfavorable RTW trajectories. In addition, tailored interventions could be initiated when unfavorable RTW trajectories are identified to improve sustainable RTW for workers with different mental disorders. Therefore, the primary aim of the present study was to investigate 2-year RTW trajectories by mental disorder diagnosis. A secondary aim was to describe the workers following the different identified RTW trajectories in terms of personal and work characteristics. Such information could provide directions for future research investigating groups of workers that may experience more difficulties in RTW and could support in further tailoring RTW interventions. Based on the available data, we examined the distribution of age, sex and contracted work hours across the diagnosis-specific RTW trajectories.

## Methods

### Study context and Dutch sickness absence management policies

In The Netherlands, workers report sick to their employer, who in turn contract an occupational health service (OHS) to register sickness absence and advice about RTW of sick-listed workers. Within six weeks of reporting sick, workers are invited for a consultation with an occupational health professional (OHP) to explore the medical, work-related and person-related factors contributing to sickness absence. Based on this consultation, the OHP gives an advice to the sick-listed worker and the employer about RTW. Commonly, the OHP advises a gradual RTW in which the amount of work hours is gradually increased to full RTW, ie, 100% of the contracted hours. The gradual increase in work hours is recorded by the employer in the OHS register as percentage partial RTW (eg, 25%, 50%, 70% of the contracted work hours). According to Dutch sickness absence policies, sick-listed workers are financially compensated by their employer for a maximum period of 24 months. Workers who fail to achieve full RTW within 24 months are assessed by the Employee Insurance Agency for a (partial) disability pension paid by the state.

### Study design and population

For this study, we used longitudinal data from the sickness absence register of a Dutch OHS providing occupational healthcare services to 1.2 million workers of approximately 12 000 large (ie, employing ≥100 workers) companies in various economic sectors throughout The Netherlands. Sickness absence (start, change and end) dates and percentages of partial RTW were retrieved from the OHS register, together with OHP sickness absence diagnoses based on the International Classification of Diseases, 10^th^ version (ICD-10) (14). Sickness absence episodes due to mental disorders diagnosed within ICD-10 chapter V (mental and behavioral disorders) and burnout (ICD-10 Z73.0) between 1 January 2013 and 31 December 2019 were used for this study. The Central Ethical Review Committee of the University Medical Center Groningen approved this study, register number 202100121.

### Study sample and data preparation

The dataset included the first day of sickness absence, dates of changes in partial RTW, and the last day of sickness absence of N=48 337 workers sick-listed with a mental disorder. The most prevalent mental disorders were: adjustment disorders (ICD-10 F43.2), anxiety disorders (ICD-10 F40-41), burnout (ICD-10 Z73.0), mood disorders (ICD-10 F30-39), and post-traumatic stress disorders and other reactions to severe stress (ICD-10 F43.1 and F43.8, from here on referred to as PTSD). For the present study, we selected workers sick-listed with these prevalent diagnoses (N=38 431; 79.5% of all workers sick-listed with a mental disorder) for the trajectory analyses. From the selected sample, we excluded employees aged ≤15 or ≥64 years at the start of sickness absence (N=580) and employees with inconsistent data, either because the sickness absence start date was after the end date (N=18) or because information about the start date of the sickness absence episode was missing (N=28). Moreover, workers who reported sick after 1 January 2018 and were still sick-listed at the end of the study period (December 2019) were excluded (N=298) because they could not be followed for the full two years. The final analytical sample included N=37 523 workers (97.0% of the total sample).

### Return-to-work (RTW)

RTW was measured as percentage of contracted work hours, with 0% RTW reflecting off-work (sickness absence) and 100% RTW reflecting full RTW, ie, working the same amount of contracted work hours as before sickness absence. For the trajectory analyses, the OHS register information was transformed into a dataset with a person-month structure, which included for every person (row) the percentage of working hours in each month (columns) from the start of the sickness absence episode until 23 months later. The RTW trajectories were followed for 23 months to prevent misclassification of workers registered administratively as 100% RTW in the 24^th^ month of sickness absence to end the sickness absence episode when the worker did not return to work and is assessed for a disability pension. Month 1 was considered the start of the sickness absence episode. If the RTW percentage changed during a month, this change was considered for the whole month. If a worker had more than one change in RTW percentage during a month, the last RTW percentage was used for that month. The RTW percentage was carried forward in the following months until the next change in RTW percentage or the end of the episode. According to Dutch sickness absence regulations, a sickness absence episode ends when a worker has 100% RTW for at least four consecutive weeks. When, in our sample, a worker called in sick more than four weeks after the end of the sickness absence episode (N=6525, ie, 13.5% of the total sample), this was regarded as a subsequent sickness absence episode. Subsequent sickness absence episodes were not analyzed in the present study because not having a subsequent sickness absence episode could also be due to the worker’s employer having left the services of the OHS or the worker having left the job. Hence, a reliable longitudinal assessment of subsequent sickness absence episodes was not possible.

### Covariates

Age, sex and contracted work hours were retrieved from the sickness absence register as covariates. Contracted work hours were categorized as fulltime (>35 hours per week), part-time (20–35 hours per week) and <20 hours per week, according to the definitions of Statistics Netherlands (15). For workers with part-time contracts, 100% RTW is achieved when they work the same number of hours per week as before reporting sick. In the Dutch labor market context, with nearly half of the working population having a part-time contract, particularly in retail, healthcare and child care (16), it is common to also consider RTW as full RTW when workers have contracts <35 hours per week.

### Statistical analyses

Latent class growth analysis (LCGA) (17) was used on the 23-month data with RTW percentages to estimate by mental disorder diagnosis a summary of RTW trajectories from the start of the sickness absence episode. LCGA identifies the most relevant types of RTW trajectory classes and provides an estimation of the percentage of the study population that can be ascribed to each identified RTW trajectory class. It needs to be taken into account that the identified trajectory classes do not necessary imply well-separated homogeneous sub-groups. Variability around the class-average trajectory is common, ie, few class members will exactly follow the average RTW trajectory and people may fit in between trajectories.

The RTW percentages were modelled with a binomial distribution (proportion of full RTW) via a logistic regression longitudinal model. We did not impose any a priori trend shape and used time as a categorical variable (monthly piecewise). Models estimated 1–15 classes. For the selection of the number of classes that reflected the best summary of RTW trajectories, we started first by looking at the model with the lowest value of the Bayesian information criterion (BIC). Then, we also explored the results of the models that had one or two classes less or more compared with the model with the lowest BIC. The final model was chosen when the additional class in the next model did not add a new type of trajectory (ie, when the new class showed a trajectory very similar to those already included in the previous model without this extra class). Each model was estimated with 250 different random starting values, and via maximum likelihood with robust standard errors. In total, we estimated five LCGA models (one for each type of mental disorder) using LatentGold version 4.5 (18).

We computed descriptives of age, sex and contracted work hours weighted by posterior class membership probabilities to explore whether descriptive patterns in the distribution of age, sex and contracted work hours appeared across the trajectories (by mental disorder diagnosis).

## Results

The final analytical sample included a total of 37 523 workers (59% women and 41% men) sick-listed with an OHP-diagnosed mental disorder between January 2013 and December 2019. Workers had a mean age of 44.3 [standard deviation (SD) 10.7] years; 49% worked fulltime, 40% 20–35 hours per week, and 11% <20 hours per week. A total of 25 075 (67%) employees were registered sick with adjustment disorders, 5076 (13%) with mood disorders, 3644 (10%) with burnout, 2393 (6%) with PTSD, and 1335 (4%) with anxiety disorders. The results of LCGA are presented in the supplementary material, www.sjweh.fi/article/4183, tables S1–S5.

### Types of RTW trajectories

**Figure 1:**
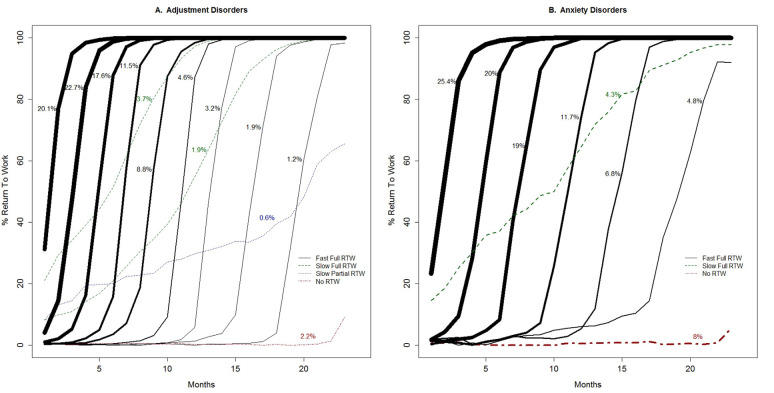
Return-to-work (RTW) trajectories among workers with adjustment disorders (A) and anxiety disorders (B); percentages refer to the class size and line thickness is proportional to the class size.

**Figure 2:**
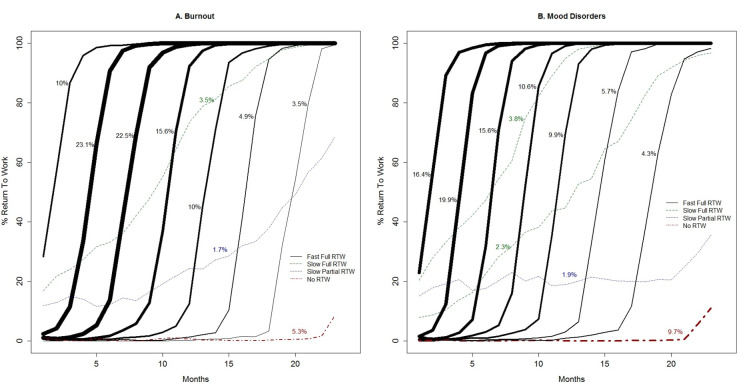
Return-to-work (RTW) trajectories among workers with burnout (A) and mood disorders (B); percentages refer to the class size and line thickness is proportional to the class size.

**Figure 3:**
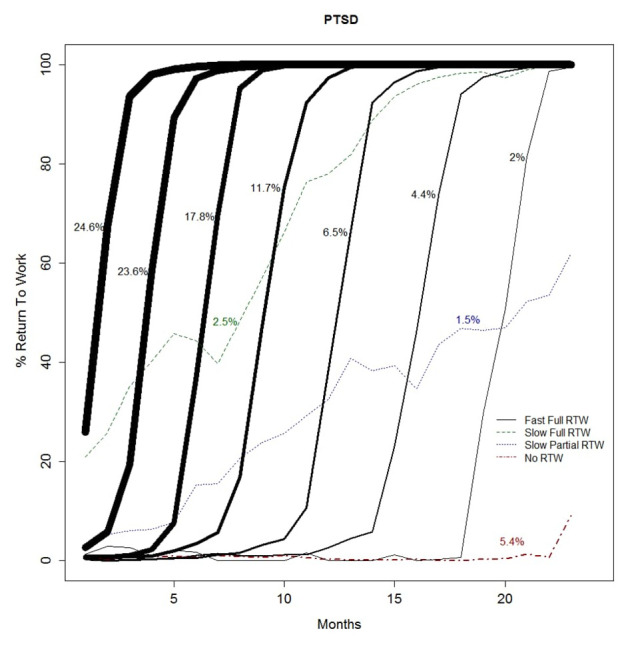
Return-to-work (RTW) trajectories among workers with post-traumatic stress disorder (PTSD); percentages refer to the class size and line thickness is proportional to the class size.

Across all mental disorder diagnoses, four different types of trajectories were identified: (i) fast full RTW, (ii) slow full RTW, (iii) slow partial RTW, and (iv) no RTW. The fast full RTW trajectories consisted of a group of sigmoidal trajectories (ie, the trajectories follow an S-shaped curve) with workers reaching 100% RTW within 3–5 months after starting RTW. Within the group of fast full RTW trajectories, the starting points of return to work varied. For most fast full RTW trajectories, the return to work started in the first 5 months of sickness absence. This subgroup of the fast full RTW trajectories was labeled ‘early-onset’ fast full RTW. For the other fast full RTW trajectories, the start of the return to work took place after 5–18 months of sickness absence. This subgroup of the fast full RTW trajectories was labeled ‘late-onset’ fast full RTW. Slow RTW trajectories showed a gradual (non-sigmoidal) increase in work hours over time, either with 100% RTW in the second sickness absence year (labeled ‘slow full’ RTW) or not reaching 100% RTW before the end of follow-up (labeled ‘slow partial’ RTW). Finally, for all mental disorder diagnoses, a group of workers established no RTW for the majority of the 23-months follow-up period. Figures 1–3 show the RTW trajectories identified for each of the five mental disorder diagnoses.

### Differences in RTW trajectories between mental disorder diagnoses

While similar types of RTW trajectories were identified across the mental disorder diagnoses, differences were found in the distribution of workers across the trajectory types. For example, when adding all sigmoidal trajectories, ie, the fast full RTW trajectories, 92.0% of the workers sick-listed with adjustment disorders followed a fast full RTW trajectory (figure 1a), while this was the case for 82.4% of the workers sick-listed with mood disorders (figure 2b). The no RTW trajectory was followed by 2.2% of the workers with adjustment disorders (figure 1a) versus 9.7% of the workers with mood disorders (figure 2b). Also, differences could be observed between mental disorder diagnoses in the proportion of workers belonging to the fast full RTW trajectories with an early onset. When adding the class sizes of the sigmoidal trajectories starting within five months after reporting sick, an approximation is derived of the proportion of workers following fast full RTW trajectory with an early onset. Workers sick-listed with adjustment disorders, anxiety disorders and PTSD (approximately 60%, 64% and 66%, see figure 1a, 1b and 3, respectively) more often belonged to the early-onset fast full RTW trajectories than workers sick-listed with burnout or mood disorders (approximately 55% and 52%, see figure 2a and 2b, respectively). A slow RTW trajectory (ending in either partial or full RTW), was the least followed trajectory for all mental disorder diagnoses, but also for this trajectory type the distribution differed between diagnoses. For the slow full RTW trajectory, the greatest difference was observed between burnout with 3.5% and mood disorders with 6.1% of the workers following this trajectory (see figures 2a and 2b). For the slow partial RTW trajectory, the greatest difference was observed between adjustment disorders with 0.6% and mood disorders with 1.6% of the workers following this trajectory (see figures 1a and 2b).

### Distribution of age, sex and working hours across the trajectories

Table 1 provides descriptive data on the distribution of age, sex and contracted work hours by type of mental disorder. A few patterns could be observed in the distribution of sex and contracted work hours across the different trajectories by mental disorder diagnosis. For all mental disorders, the percentage of women increased for the fast full RTW trajectories with later onsets of RTW. For example, of the workers with adjustment disorders following the fast full RTW trajectory with the earliest RTW onset 52.7% were women, while for the fast full RTW trajectory with the latest RTW onset this was 72.0%. For mood and anxiety disorders, the trajectories with no RTW were characterized by the highest percentages of women compared with all other trajectories.

**Table 1:** Distribution of covariates across the return-to-work (RTW) trajectories per mental disorder diagnosis. [PTSD=post-traumatic stress disorder.]

Class (%) ^1^		Age (mean, SE)	Work hours (%)				Sex (%)	
			<20	20–35	>35		Female	Male
Adjustment disorders ^2^								
	1 (20.1)	44.2 (0.1)	10.0	36.8	53.2		52.7	47.3
	2 (22.7)	44.3 (0.1)	10.7	38.8	50.5		56.7	43.3
	3 (17.6)	44.6 (0.1)	10.2	40.5	49.3		60.3	39.7
	4 (11.5)	44.7 (0.2)	10.5	42.1	47.5		64.2	35.8
	5 (8.8)	44.9 (0.2)	10.9	42.7	46.4		64.6	35.4
	6 (4.6)	45.4 (0.3)	11.0	42.9	46.1		66.0	34.0
	7 (3.2)	45.6 (0.3)	10.1	45.9	44.0		69.7	30.3
	8 (1.9)	46.6 (0.4)	11.2	44.0	44.8		71.5	28.5
	9 (1.2)	45.8 (0.6)	13.9	44.4	41.7		72.0	28.0
	10 (3.7)	45.0 (0.2)	6.1	42.5	51.5		60.9	39.1
	11 (1.9)	45.6 (0.4)	7.3	43.4	49.3		61.5	38.5
	12 (0.6)	44.3 (0.8)	9.1	52.3	38.6		63.1	36.9
	13 (2.2)	46.2 (0.5)	11.4	49.1	39.5		71.2	28.8
Anxiety disorders ^3^								
	1 (25.4)	39.1 (0.6)	19.8	30.0	50.3		52.1	47.9
	2 (20.0)	40.3 (0.6)	15.2	39.0	45.8		57.6	42.4
	3 (19.0)	40.6 (0.6)	11.5	43.6	44.9		61.7	38.3
	4 (11.7)	40.3 (0.8)	10.8	42.5	46.7		61.8	38.2
	5 (6.8)	39.3 (1.1)	17.0	40.2	42.7		63.0	37.0
	6 (4.8)	40.6 (1.1)	12.4	44.3	43.2		66.0	34.0
	7 (4.3)	42.5 (1.1)	7.3	39.0	53.7		51.7	48.3
	8 (8.0)	40.7 (1.1)	26.9	37.0	36.0		69.1	30.9
Burnout ^4^								
	1 (10)	44.7 (0.5)	7.6	32.9	59.6		51.9	48.1
	2 (23.1)	44.9 (0.3)	7.7	34.9	57.4		52.9	47.1
	3 (22.5)	44.7 (0.3)	7.2	36.5	56.3		55.9	44.1
	4 (15.6)	45.6 (0.4)	6.7	38.2	55.1		59.0	41.0
	5 (10.0)	46.5 (0.5)	7.4	39.9	52.7		63.1	36.9
	6 (4.9)	47.2 (0.7)	6.7	38.2	55.1		59.0	41.0
	7 (3.5)	47.2 (0.9)	16.5	38.6	44.9		65.4	34.6
	8 (3.5)	44.9 (0.7)	2.8	40.6	56.6		60.9	39.1
	9 (1.7)	46.6 (1.2)	8.9	46.5	44.6		69.4	30.6
	10 (5.3)	47.0 (0.8)	10.1	40.5	49.4		65.3	34.7
Mood disorders ^5^								
	1 (16.4)	43.0 (0.4)	15.7	33.7	50.6		47.0	53.0
	2 (19.9)	42.8 (0.3)	15.8	37.0	47.2		50.2	49.8
	3 (15.6)	43.0 (0.4)	13.1	34.1	52.8		49.5	50.5
	4 (10.6)	43.6 (0.4)	12.2	38.8	49.1		57.2	42.8
	5 (9.9)	43.6 (0.5)	14.6	37.8	47.7		55.9	44.1
	6 (5.7)	44.9 (0.6)	10.5	42.3	47.2		57.3	42.7
	7 (4.3)	44.4 (0.7)	13.9	41.5	44.6		57.5	42.5
	8 (3.8)	43.7 (0.6)	6.8	36.5	56.7		50.8	49.2
	9 (2.3)	45.3 (0.8)	5.9	45.1	48.9		56.4	43.6
	10 (1.9)	45.7 (1.0)	6.7	51.3	42.1		48.8	51.2
	11 (9.7)	45.9 (0.5)	13.8	38.7	47.5		60.9	39.1
PTSD ^6^								
	1 (24.6)	42.8 (0.4)	7.9	34.1	58.0		50.0	50.0
	2 (23.6)	41.9 (0.4)	9.7	37.9	52.4		52.7	47.3
	3 (17.8)	43.0 (0.5)	11.4	39.2	49.4		57.1	42.9
	4 (11.7)	44.1 (0.6)	9.0	43.5	47.5		61.1	38.9
	5 (6.5)	44.9 (0.8)	11.4	40.0	48.6		67.7	32.3
	6 (4.4)	42.9 (1.0)	11.1	39.7	49.2		63.1	36.9
	7 (2.0)	45.2 (1.6)	14.9	30.7	54.4		45.9	54.1
	8 (2.5)	43.7 (1.1)	4.4	37.2	58.4		61.4	38.6
	9 (1.5)	45.2 (1.6)	10.1	35.3	54.6		56.6	43.4
	10 (5.4)	44.0 (0.9)	10.2	45.0	44.8		57.9	42.1

^1^ For each class, the class size as percentage of the study population is presented. ^2^ Class 1–9: fast full RTW trajectories (the higher the class number the later the starting point of RTW); Class 10–11: slow full RTW trajectory; Class 12: slow partial RTW trajectory; Class 13: no RTW trajectory. ^3^ Class 1–6: fast full RTW trajectories (the higher the class number the later the starting point of RTW); Class 7: slow full RTW trajectory; Class 8: no RTW trajectory. ^4^ Class 1-7: fast full RTW trajectories (the higher the class number the later the starting point of RTW); Class 8: slow full RTW trajectory; Class 9: slow partial RTW trajectory; Class 10: no RTW trajectory. ^5^ Class 1-7: fast full RTW trajectories (the higher the class number the later the starting point of RTW); Class 8-9: slow full RTW trajectory; Class 10: slow partial RTW trajectory; Class 11: no RTW trajectory. ^6^ Class 1-7: fast full RTW trajectories (the higher the class number the later the starting point of RTW); Class 8: slow full RTW trajectory; Class 9: slow partial RTW trajectory; Class 10: no RTW trajectory.

For all mental disorders, the percentage of workers on full-time contracts (ie, >35 hours) decreased for the fast full RTW trajectories with later onsets of RTW. In the no RTW trajectory, lower percentages of workers had full-time contracts compared with the fast full RTW trajectories with earlier starting points of RTW. The slow full RTW trajectories were characterized by the lowest percentages of workers with <20-hour work contracts compared with all other trajectories. No age patterns were observed across the different RTW trajectories.

## Discussion

To the best of our knowledge, this is the first study investigating RTW trajectories by mental disorder diagnosis. Our results provide new insights into the RTW process of workers sick-listed with mental disorders. We showed that RTW trajectories are very similar for the most prevalent mental disorder diagnoses in the Dutch OHS context, but also identified differences between diagnoses with regard to the *distribution* of workers across the RTW trajectories. For example, the percentages of workers following a slow or no RTW trajectory were highest for mood disorders (eg, 9.7% of the workers with mood disorders followed a no RTW trajectory versus 2.2% of the workers with adjustment disorders). Unlike other studies (11–13), we identified different times of onset of fast full RTW trajectories, ie, some workers had an early onset of RTW within five months after reporting sick, while other workers had a later onset but a similar fast increase in work hours until full RTW. The workers with a late onset fast full RTW trajectory accumulate a high number of sickness absence days. Workers with a slow RTW trajectory also accumulate a high number of sickness absence days, though they constitute a different group. When only considering the total number of sickness absence days, these two different groups cannot be distinguished. It is important, however, to differentiate between workers with a late onset fast full RTW trajectory versus a slow RTW trajectory because their gradual RTW process is very different and workers may need different types of support or interventions to facilitate RTW.

For all mental disorder diagnoses, the majority of workers followed a fast full RTW trajectory (ranging from 82.4% for mood disorders to 92.0% for adjustment disorders). The distribution of workers across fast full RTW trajectories with a different onset of RTW varied between mental disorder diagnoses. For example, a higher percentage of workers with adjustment disorders followed fast full RTW trajectories with an onset within five months after reporting sick compared with workers with mood disorders. It is conceivable that RTW trajectories start earlier when workers experience mild or moderate symptoms and later if workers experience a high symptom burden or a comorbid disorder. A scoping review of 2447 studies showed that among workers with mental disorders, earlier RTW is consistently predicted by lower symptom severity, having no previous absenteeism, younger age and positive expectations concerning sick leave duration or RTW (19). A recent longitudinal qualitative study also provides potential explanations for the differences in times to onset of fast full RTW trajectories (20). Joosen et al identified three main differences between workers with short-term versus long-term sickness absence, respectively: (i) favorable versus unfavorable work conditions (eg, good versus poor relationship with the supervisor and colleagues); (ii) pro-active versus reactive recovery behavior (eg, actively taking recovery actions versus relying on health care providers); and (iii) enjoying many aspects of work versus experiencing work as less fulfilling over the years (20).

We explored differences in the distribution of age, sex and work hours across the RTW trajectories by mental disorder diagnosis and found similar patterns across diagnoses. Compared with the fast full RTW trajectories with an early onset, the fast full RTW trajectories with a later onset of RTW comprised more women and fewer workers with full-time contracts. Future research is needed to further identify characteristics of both the worker and the work environment that discern workers in the different trajectories to provide entry points for tailored RTW support.

### Strengths and limitations

The large number of included workers covering all economic sectors adds to the generalizability of the study results within The Netherlands and increases the likelihood that we identified all possible RTW trajectory trends within the studied population. However, the fact that the study only included workers employed in large companies (>100 workers) implies that the results may not apply to small and medium-sized enterprises. Furthermore, our findings might not generalize to other countries with different sickness absence policies and practices. The use of OHS sickness absence register data is a strength of the study, avoiding workers’ recall and information bias of self-reported sickness absence and RTW. A further strength is the use of OHP-diagnosed mental disorders instead of self-reported symptoms. Although the validity of OHP diagnoses is debatable (21), Hoogduin & Van Leusden (22) found an 81% agreement between Dutch OHPs and psychiatrists on mental causes for sickness absence. An important limitation is the restricted number of variables representing characteristics of workers and their workplaces in the OHS sickness absence register, a known limitation of sickness absence register studies. Hence, a detailed investigation of why workers started RTW later or followed partial or no RTW trajectories was not possible. Another limitation of the OHS sickness absence register is that only one diagnosis can be reported. Hence, information on comorbidity was not available. As mental disorders frequently co-exist (23), similarities in RTW trajectories between different disorders may be partly explained by comorbidity. Still, we did identify some differences in RTW trajectory types and the distribution of workers across trajectories between the five mental disorder groups. Investigating worker groups without comorbid mental disorders may result in different findings with regards to similarities and differences in RTW trajectory types between mental disorder diagnoses. Finally, we dealt with potential misclassification due to RTW being administratively set at 100% in the 24^th^ month of sickness absence when employment ends and the sick-listed worker is assessed for a disability pension, by choosing a 23-month follow-up period. RTW is also administratively set at 100% when employment ends during sickness absence for other reasons, like a temporary contract. We assume that these other reasons for ending employment do not differ across mental disorder diagnoses. Furthermore, in The Netherlands, employers cannot fire workers during periods of sickness absence. Hence, we do not expect differential misclassification due to RTW administratively set at 100% when employment ends during sickness absence.

### Practical implications and directions for future research

As RTW trajectories were very similar for different types of mental disorder diagnoses, the OHP diagnosis of the type of mental disorder may not be an appropriate entry point for interventions to support RTW. This finding fits the recently developed network theory of mental disorders, which assumes that symptoms of psychopathology are connected through myriads of biological, psychological and societal mechanisms that are more important than DSM or ICD diagnostic categories (24). Hence, a transdiagnostic approach, focusing on therapeutic elements and interventions effective for various types of mental disorders, may be recommended (25, 26). OHPs should timely identify the workers at risk of a late start of RTW, slow RTW, and no RTW, regardless of the type of mental disorder. For that purpose, we need more research into the risk factors for a late onset, slow or no RTW trajectory to better tailor interventions that facilitate RTW. For example, support and interventions may be different for workers with late onset fast full RTW compared with workers with slow RTW trajectories. Moreover, future research should focus on the associations between different RTW trajectories and recurrent mental health-related sickness absence. It would be particularly interesting to investigate whether workers with a late onset fast RTW trajectory have a different risk for recurrent mental health-related sickness absence compared with workers with a slow RTW trajectory.

### Concluding remarks

We found that RTW trajectories were similar for workers sick-listed with adjustment disorders, anxiety disorders, burnout, mood disorders, and PTSD, and we identified a fast full, slow full, slow partial, and no RTW trajectory. The distribution of workers across RTW trajectories differed between mental disorder diagnoses. A transdiagnostic approach addressing factors that delay the onset of RTW or contribute to slow or no RTW, could support workers sick-listed with mental disorders in their RTW.

## Supplementary material

Supplementary material
