# Supplementary material for: Do return-to-work trajectories differ by mental disorder diagnosis? A register study among 37 523 Dutch workers
Source: Scand J Work Environ Health. 2024 Sep 30;50(7):527–35. doi: 10.5271/sjweh.4183 (PMC11473124; doi:10.5271/sjweh.4183)
Supplement: Supplementary material [file SJWEH-50-527-S001.pdf]

Do return-to-work trajectories differ by mental disorder diagnosis? A register study among 37 523 Dutch workers[1]

by Robèrt Vendelbosch, MSc, Corné Roelen, PhD, Josué Almansa, PhD, Ute Bültmann, PhD, Iris Arends, PhD [2]

1. Supplementary material
2. Correspondence to: Iris Arends, PhD, University of Groningen, University Medical Center Groningen, Department of Health Sciences, Community & Occupational Medicine, Hanzeplein 1, Postbus 30.001, 9700 RB, Groningen, the Netherlands. [E-mail: i.arends@umcg.nl]

Results of the Latent Class Growth Analyses

For adjustment disorders, anxiety disorders, burnout, mood disorders and PTSD, 13, 8, 10, 11 and 10 classes showed the best model fit, respectively (see Tables S1, S2, S3, S4 and S5). For PTSD, the fit indices of the 8 and 9 class model were very similar (see Table S5). The 9-class model was preferred because it included a new type of trajectory: a slow full RTW trajectory. Comparing the 9-class model with the 10-class model, there was one more gradation of the fast full RTW trajectory, and for the no RTW trajectory the maximum RTW percentage was lower lying around 10% RTW instead of 20% RTW, i.e. more in line with our classification of 'no RTW'. The size of the no RTW trajectory decreased with 1% from 6% to 5% of the study population with this 1% moving to the fast full RTW trajectory. We assumed that the 10-class model better fits reality, because in this model the no RTW trajectory seems more truly a non-recovery class as it includes less people who actually return to work (as compared with the 9-class model).

Table S1. Fit indices for the latent class growth model estimating the number of RTW trajectories for adjustment disorders. [LL=log likelihood (higher values imply better fit); BIC=Bayesian information criterion (the model with the lowest value is preferred); Npar=number of parameters]

Number of classes	LL	BIC	Npar	Entropy[a]
1	-199032,9	398298,8	23	
2	-133699,9	267875,8	47	0,954
3	-111813,4	224346,1	71	0,927
4	-102216,4	205395,1	95	0,897
5	-98283,6	197772,5	119	0,870
6	-95981,6	193411,7	143	0,856
7	-94798,9	191289,4	167	0,860
8	-93687,4	189309,6	191	0,831
9	-93107,0	188392,0	215	0,841
10	-92553,2	187527,4	239	0,814
11	-92233,4	187130,8	263	0,798
12	-91984,0	186875,2	287	0,795
**13[b]**	**-91803,3**	**186756,9**	**311**	**0,765**
14	-91765,3	186924,0	335	0,792
15	-91595,7	186828,0	359	0,760

[a]Entropy refers to the quality of class assignment (a higher entropy implies better accuracy of class assignment)

[b]In bold is the selected number of classes.

Table S2. Fit indices for the latent class growth model estimating the number of RTW trajectories for anxiety disorders. [LL=log likelihood (higher values imply better fit); BIC=Bayesian information criterion (the model with the lowest value is preferred); Npar=number of parameters]

Number of classes	LL	BIC	Npar	Entropy[a]
1	-14373,5	28912,6	23	
2	-8934,9	18208,1	47	0,959
3	-7162,9	14836,7	71	0,951
4	-6404,8	13493,3	95	0,934

5	-6112,5	13081,4	119	0,920
6	-5962,7	12954,4	143	0,898
7	-5848,9	12899,5	167	0,900
**8[b]**	**-5761,5**	**12897,4**	**191**	**0,889**
9	-5717,4	12982,0	215	0,883
10	-5682,7	13085,3	239	0,877
11	-5654,0	13200,6	263	0,867
12	-5624,8	13314,8	287	0,856
13	-5604,5	13446,9	311	0,855
14	-5594,5	13599,6	335	0,855
15	-5580,0	13743,3	359	0,815

[a]Entropy refers to the quality of class assignment (a higher entropy implies better accuracy of class assignment)

[b]In bold is the selected number of classes.

Table S3. Fit indices for the latent class growth model estimating the number of RTW trajectories for burnout. [LL=log likelihood (higher values imply better fit); BIC=Bayesian information criterion (the model with the lowest value is preferred); Npar=number of parameters]

Number of classes	LL	BIC	Npar	Entropy[a]
1	-36097,7	72384,1	23	
2	-23854,6	48094,7	47	0,958
3	-19602,9	39788,0	71	0,943
4	-17874,3	36527,7	95	0,925
5	-17055,9	35087,8	119	0,906
6	-16677,6	34527,8	143	0,879
7	-16363,8	34097,1	167	0,877
8	-16068,1	33702,5	191	0,881
9	-15917,2	33597,7	215	0,860
**10[b]**	**-15814,1**	**33588,2**	**239**	**0,870**
11	-15750,6	33658,0	263	0,867
12	-15685,5	33724,6	287	0,847
13	-15644,3	33839,1	311	0,826

14	-15594,3	33935,9	335	0,816
15	-15566,1	34076,2	359	0,804

[a]Entropy refers to the quality of class assignment (a higher entropy implies better accuracy of class assignment)

[b]In bold is the selected number of classes.

Table S4. Fit indices for the latent class growth model estimating the number of RTW trajectories for mood disorders. [LL=log likelihood (higher values imply better fit); BIC=Bayesian information criterion (the model with the lowest value is preferred); Npar=number of parameters]

Number of classes	LL	BIC	Npar	Entropy[a]
1	-56587,1	113370,3	23	
2	-34305,1	69011,2	47	0,975
3	-27139,5	54884,8	71	0,955
4	-24785,7	50381,9	95	0,931
5	-23420,0	47855,4	119	0,923
6	-22857,4	46934,9	143	0,900
7	-22380,8	46186,4	167	0,900
8	-21997,7	45625,1	191	0,887
9	-21747,9	45330,2	215	0,887
10	-21586,6	45212,3	239	0,864
**11[b]**	**-21458,0**	**45159,9**	**263**	**0,862**
12	-21364,0	45176,8	287	0,861
13	-21307,8	45269,2	311	0,861
14	-21259,2	45376,8	335	0,841
15	-21221,3	45505,7	359	0,842

[a]Entropy refers to the quality of class assignment (a higher entropy implies better accuracy of class assignment)

[b]In bold is the selected number of classes.

Table S5. Fit indices for the latent class growth model estimating the number of RTW trajectories for PTSD. [LL=log likelihood (higher values imply better fit); BIC=Bayesian information criterion (the model with the lowest value is preferred); Npar=number of parameters]

Number of classes	LL	BIC	Npar	Entropy[a]

1	-23625,8	47430,6	23	
2	-14580,8	29527,2	47	0,973
3	-11775,6	24103,5	71	0,952
4	-10630,2	21999,5	95	0,938
5	-10128,2	21182,2	119	0,909
6	-9839,2	20790,9	143	0,892
7	-9655,2	20609,7	167	0,895
8	-9517,2	20520,4	191	0,870
9	-9427,2	20527,2	215	0,869
**10**[b]	**-9340,4**	**20540,3**	**239**	**0,870**
11	-9286,8	20619,8	263	0,851
12	-9256,1	20745,2	287	0,848
13	-9232,6	20884,8	311	0,844
14	-9214,3	21034,9	335	0,821
15	-9192,5	21178,2	359	0,820

[a]Entropy refers to the quality of class assignment (a higher entropy implies better accuracy of class assignment)

[b]In bold is the selected number of classes.
